# Natural Korean Medicine Dang-Gui: Biosynthesis, Effective Extraction and Formulations of Major Active Pyranocoumarins, Their Molecular Action Mechanism in Cancer, and Other Biological Activities

**DOI:** 10.3390/molecules22122170

**Published:** 2017-12-07

**Authors:** Chinreddy Subramanyam Reddy, Seong Cheol Kim, Mok Hur, Yeon Bok Kim, Chun Geon Park, Woo Moon Lee, Jae Ki Jang, Sung Cheol Koo

**Affiliations:** Department of Herbal Crop Resources, National Institute of Horticultural & Herbal Science, RDA, Eumseong-gun 27709, Korea; suumani@gmail.com (C.S.R.); kimsec@korea.kr (S.C.K.); mok0822@korea.kr (M.H.); yeondarabok@korea.kr (Y.B.K.); pcg@korea.kr (C.G.P.); wmlee65@korea.kr (W.M.L.); changjk@korea.kr (J.K.J.)

**Keywords:** natural medicine, Korean dang-gui, apoptosis, cancer, decursin

## Abstract

*Angelica gigas* Nakai (AGN) is a crucial oriental medicinal herb that grows especially in Korea and the Far-East countries. It contains chemically active compounds like pyranocoumarins, polyacetylenes and essential oils, which might be useful for treatment of several chronic diseases. It has been used for centuries as a traditional medicine in Southeast Asia, but in Western countries is used as a functional food and a major ingredient of several herbal products. The genus *Angelica* is also known as ‘female ginseng’ due to its critical therapeutic role in female afflictions, such as gynecological problems. However, it is well-documented that the AGN pyranocoumarins may play vital beneficial roles against cancer, neurodisorders, inflammation, osteoporosis, amnesia, allergies, depression, fungi, diabetes, ischemia, dermatitis, reactive oxygen species (ROS) and androgen. Though numerous studies revealed the role of AGN pyranocoumarins as therapeutic agents, none of the reviews have published their molecular mechanism of action. To the best of our knowledge, this would be the first review that aims to appraise the biosynthesis of AGN’s major active pyranocoumarins, discuss effective extraction and formulation methods, and detail the molecular action mechanism of decursin (D), decursinol angelate (DA) and decursinol (DOH) in chronic diseases, which would further help extension of research in this area.

## 1. Introduction

*Angelica gigas* (local name dang-gui) is also well-known as female ginseng due to its favorable curing properties in female afflictions, and it has been used for centuries in Far East countries [1,2]. The name Angelica was derived from a European mythological belief, which states that in the Middle Ages, while Europe was ruined by the plague disease, apparently, an angel came and presented the herb to a monk to cure the disease. Since then, the herb is renowned by the name of Angelica and it can be found in different archaic literature. It is a biannual or short-lived perennial and an Oriental traditional medicinal herb, also known as dang-gui locally. The natural habitat of this plant is mainly fens, riverbanks, damp meadows and woods. It requires rich, moist and well-drained soil under partial shade conditions, and can grow up to eight feet tall [3]. More than 60 *Angelica* species are present around the world, contributing the highest source of pyranocoumarins. *Angelica gigas* Nakai (AGN) mostly occurs in East Asia, especially Korea. 

The extract of the dried root of AGN (cham dang-gui) has crucial medicinal properties. The vital chemical substances having medicinal value are pyranocoumarins, furocoumarins, coumarin, phenolics, phthalides, polysaccharides and volatile compounds [4]. In the alcohol extraction, major chemical substances are isolated from cham dang-gui ; pyranocoumarins that include decursin (D) (18.7–44.8 mg/g), decursinol angelate (DA) (11.1–36.8 mg/g), nodakenin (4.53–13.1 mg/g), demethylsuberosine (0.412–2.31 mg/g), decursinol (DOH) (0.083–0.431 mg/g) and marmesin (0.059–0.592 mg/g) [5]. Several research publications have documented anticancer and anti-inflammatory properties, and neuroprotective abilities, of AGN coumarins [6,7,8,9,10,11,12,13,14,15,16,17,18,19,20]. Biosynthesis of pyranocoumarins including decursin, decursinol angelate and decursinol takes place through the phenylpropanoid pathway in plants. The most abundant pyranocoumarin, decursin, was first isolated from the *A. decursiva* root; later, in 1960, decursin was extracted from AGN [4]. Ethanol- or methanol-extracted coumarins from AGN exhibited potential effects against various types of cancers and neurorelated problems. Decursin and decursinol angelate are abundantly present in AGN, whereas the core compound decursinol is found in very low quantity. In contrast to the Chinese and Japanese dang gui, Korean dang-gui (AGN) root has an abundant D and DA content [21]. 

Several studies have revealed the significant role of D and its isomer DA in curing cancer and neurological disorders. In-vitro and in-vivo studies with human cells and mice model systems have confirmed the potential role of these compounds in combating deadly diseases like cancer and neuronal degeneration. Also, the roles of D and DA in programmed cell death (apoptosis) in cancer and neurodisordered cells have been studied to a large extent. There are two types of apoptosis processes: mitochondrial-dependent and independent apoptosis. So far, numerous reports have been published regarding AGN’s therapeutic applications in cancer and neurologically related problems; however, very limited information underlying molecular mechanisms is available. Hence we have reviewed the molecular action mechanism of D’s and DA’s therapeutic role in chronic diseases. This review summarizes *Angelica gigas* Nakai’s active chemical compounds’ (AGNACCs’) biosynthesis, their efficient extraction and formulation methods, and their underlying mechanism for chronic diseases.

## 2. Biosynthesis of Major AGNACCs D, DA and DOH

**Figure 1:**
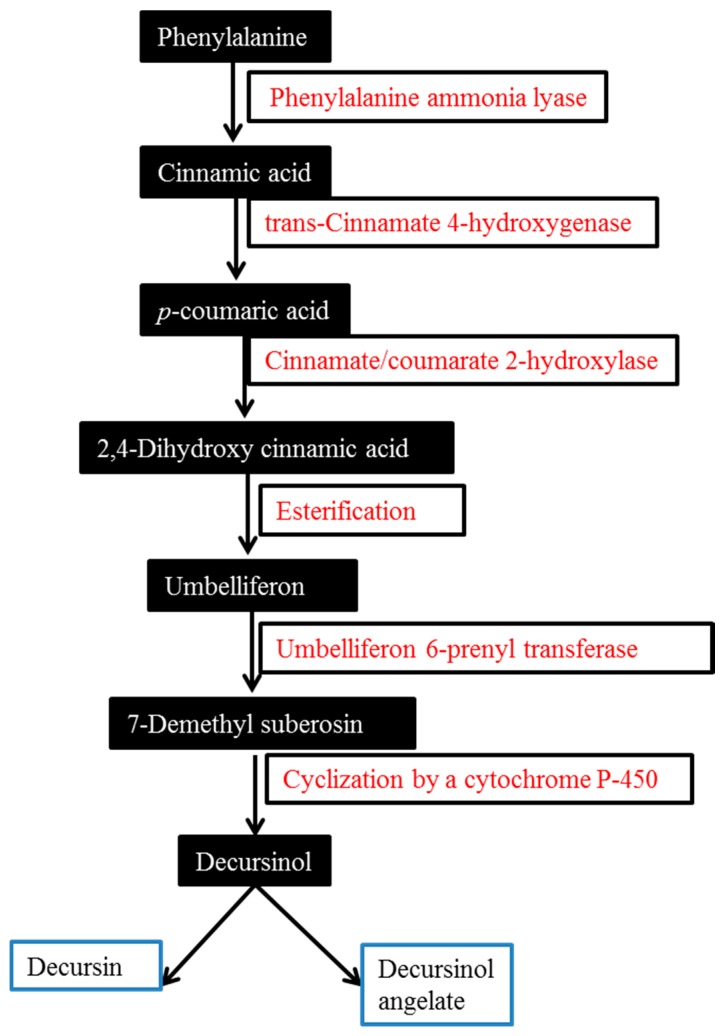
Biosynthesis of decursinol and its derivatives decursin and decursinol angelate in *Angelica gigas* Nakai root.

As we mentioned earlier, in roots, D and DA are present in abundance as compared to DOH. Biosynthesis of these active chemical compounds is a result of the phenylpropanoid pathway (Figure 1), where phenylalanine serves as a precursor for the accumulation of secondary metabolites such as flavonoids, anthocyanin and carotenoids [22]. The amino acid phenylalanine gets converted into cinnamic acid in the presence of phenylalanine ammonia lyase (PAL), and further hydroxylation takes place at C-3 in the benzene ring through the action of *trans*-cinnamate 4-hydroxygenase (C4H), which generates *p*-Coumaric acid. *p*-Coumaric acid is further hydroxylated at C-2 by the action of cinnamate-2 hydroxylase and is converted into umbelliferon by (nonenzymatic) esterification. Through the action of umbelliferon 6 prenyl transferase, umbelliferon turns into dimethylsuberosin and ultimately it gets converted into decursinol by the action of cytochrome P-450. The mechanism of the last step of the biosynthetic pathway has not yet been fully understood [23]. A study by Ji et al. involving deuterium-labeled isotope data supported this phenylpropanoid pathway in AGN hairy root culture. PAL and C4H are crucial enzymes in this pathway that are useful to convert phenylalanine to cinnamic acid and *p*-coumaric acid, respectively. The amount of these enzymes in AGN’s fine roots is the high [24]. Hence, these enzymes were overexpressed in AGN hairy roots by Park et al. and their results showed improved DA content in transgenic hairy roots in C4H lines [25].

## 3. Extraction of AGNACCs and Effective Formulation Methods

Plant pharmacological products can be prepared by extraction, grinding-filtration and freeze-drying methods; however, these approaches are labor intensive, time consuming and expensive. AGN’s natural pharmacological products are commonly extracted with alcohol [26] and hot water [27] as extraction solvents. AGNACCs D, DA and DOH are hydrophobic in nature and poorly solubilize in water, thus, diverse physical and chemical methods are employed for extraction of these compounds. Different methods and formulations have been exploited to enhance the extraction of therapeutically active compounds [28,29]. A colloidal diffusion with nanoparticles method was utilized to extract these compounds for oral administration in a therapeutic study [30]. Microemulsions (MEs) are extensively employed to enhance the hydrophilicity and mucosal absorption of drugs. Their favorable properties such as the intact nature of the solution and thermodynamic stability of MEs is widely accepted [31,32]. Ethanol-extracted AGN (AGNEtOH) yields decursin and decursinol angelate which were screened to elucidate their therapeutic potential by using omega-3 (ω-3)-based ME systems. The chemical substances greatly dissolved in the ω-3 mixture, and approximately 10- and 6-times higher dissolution was possible as compared to water [33]. The same group developed another formulation to extract the AGN root derivatives for oral delivery. They used nanoparticles for oral administration. The formulation included amphiphilic copolymer Soluplus^®^ mixed with AGNEtOH, and an electro-hydrodynamic method was followed. The pharmacokinetics results in mice exhibited greatly improved oral absorption as compared to the ethanol-extracted AGN derivatives [5].

Among various methods, hot water extrusion (HWE) is desirable for hydrophobic compounds [34] due to its favorable properties. AGN powder’s particle size also influences the treatment of menopause. Ultrafine powder exhibited better pharmacological bioactivity [6]. AGN ultrafine powder refined with a combination of physical (HME) and chemical (Soluplus^®^) modification significantly improved solubility of AGNACCs. The preparative mix, when orally administered to the scopolamine, induced amnesia in rats, greatly ameliorated than AGNEtOH. To conclude, this method could be more effective than other methods, as this method results in high solubility of AGNACCs [28].

## 4. AGNACCs and Their Role in Major Biological Activities 

**Figure 2:**
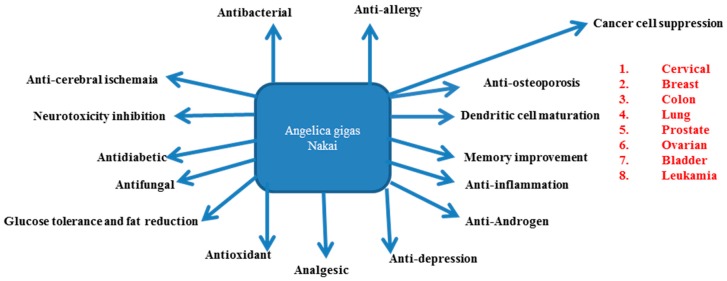
*Angelicas gigas* Nakai’s active chemical compounds’ major biological activities.

As we mentioned earlier, AGN is the finest source to pursue research on D and DA due to a higher quantity of pyranocoumarins than Chinese (*A. sinensis*) and Japanese (*A. acutiloba*) dang-gui [35]. Though several chemical compounds exist in AGN root, D, DA and DOH are considered as vital active compounds due to their favorable medical applications in the treatment of diseases. The major roles of AGN’s active chemical compounds in several biological activities are depicted in Figure 2. In addition, decursin and/or DA were reported in some herbal formulations such as ka-mi-kae-kyuk-tang (KMKKT) [36], bangpungtongsung-san [37], sanghwa tang (SHT) [38], LMK02-jangwonhwan [39], sipjundaebo-tang [40] and ojeok-san (OJS) [37].

## 5. AGNACCs’ Pharmacokinetics in Rodents and Humans

It is well-documented that D and DA expeditiously change into decursinol in rodents after feeding [9,20,41,42]. In-vitro experiments in human and murine liver microsomal bodies revealed that various enzymes metabolize D and DA into DOH. DA is converted into DOH specifically in the presence of cytochrome P-450 [20]. In the case of D, cytochrome isoform CYP2C19 and carboxylesterase-2 are required. It is a well-known fact that the conversion process of D and DA into DOH takes place in the liver; however, to identify the metabolic differences in humans and rodents, the same group executed experiments with human and rodent liver S9 fractions. The in-vitro results demonstrated that the human liver S9 fraction more-slowly metabolized D and DA into DOH than rodents, and concluded that humans and rodents may follow different mechanisms in D and DA conversion processes [43]. Furthermore, the same group pursued research to understand the varied mechanisms of the DOH metabolizing process by a pharmacokinetics study in humans by giving a single oral dose of D and DA [44]. The study found no negative effects on Caucasians as well as Hispanics, which is encouraging for cancer research progression with AGN extracts. They gave four Vegicaps^®^ with 119 mg of D and 77 mg of DA to 20 people, analyzed their plasma using HPLC–MS/MS and identified that men metabolized faster than women. They found that the mean time (T_max_) was 2.1, 2.4 and 3.3 h, and that the peak maximum concentration was 5.3, 48.1 and 2480 nmol/L, for D, DA and DOH, respectively. The half-life of D, DA and DOH was 17.4, 19.3 and 7.4 h, respectively, and the authors also elucidated that the human plasma DOH C_max_ value was comparable to rodents, whereas the DA C_max_ value was seven-times higher. Hence, DA and its isomer D were metabolized more-slowly than DOH. However, the metabolic fate is qualitatively quite similar in humans and rodents.

## 6. AGNACCs’ Molecular Action Mechanism in Cancer

### 6.1. Basic Principle of AGNACCs’ Action Mechanism in Cancer and Other Biological Activities

**Figure 3:**
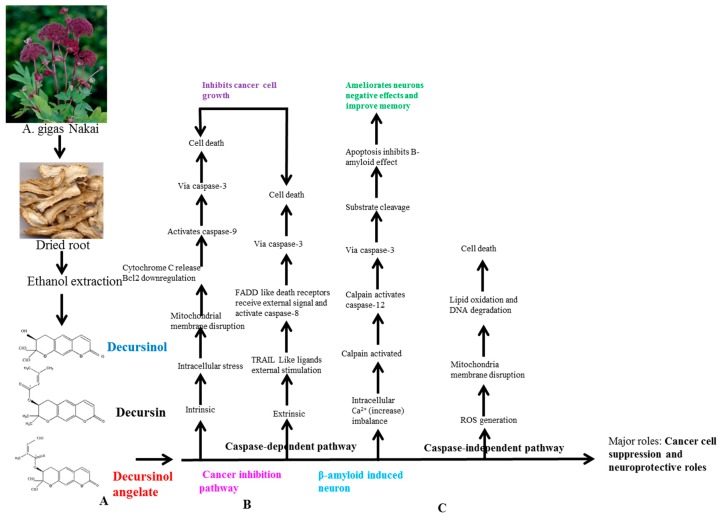
*Angelica gigas* Nakai’s root extract’s major pyranocoumarins’ molecular action mechanism in cancer and neurodisorders. (**A**) AGN’s major active pyranocoumarins’ molecular structures; (**B**) Cancer inhibition pathways by cysteine proteases, caspase-dependent and -independent molecular action mechanisms; (**C**) Nervous system damage control by programmed cell death (apoptosis).

Unusual cell proliferation spreading to other parts of the body is well known as cancer. Millions of people are affected by cancer in the world, and in 2015, 90.5 million people had it [45]. A few methods are available for curing cancer, like chemotherapy, immunotherapy and irradiation. However, these methods have severe side effects. Hence, there is a great demand for natural remedies, especially with phytochemicals [36]. Several attempts were made by using the AGNACCs, and it was found that they are effective at suppressing cancer cells through programmed cell death. The programmed cell death, also known as apoptosis, is a biochemical modification that leads to a morphological change in the cells, and ultimately it causes cell death [46]. Apoptosis is considered a protective mechanism to eradicate catastrophic cells. Apoptosis is indicated by apoptotic bodies’ formation, chromatin condensation, breakage in DNA, and G1-phase cell-cycle arrest. Programmed cell death induction is a very attractive method in cancer treatment. Apoptosis can be activated by two major signaling pathways. Intrinsic apoptosis signals are generated within the cell by mitochondria, whereas extrinsic apoptosis is induced by external death ligands like TRAIL and FADD [47]. These signals activate cysteine proteases (caspases), and via caspase-9, -12 and -3, the apoptosis process leads to cell death. Even without involvement of caspases, the process of programmed cell death completes in what is also known as the caspase-independent pathway (The whole cycle depicted in Figure 3). In addition to cancer therapy, the apoptosis process is useful in many other diseases, such as neurodegenerative diseases, diabetes, stroke and so on.

### 6.2. AGNACCs Action Mechanism in Cervical Cancer

Cervical cancer is one of the severe malignancies in women and it holds 8th place in mortality among cancers. It is usually treated with cisplatin/cisplatin-based chemotherapy, which have a number of side effects; hence, there is huge concern to focus on anticancer-related natural remedies [47,48]. Yim et al. [49] screened five chemical compounds from AGN extract, including D, DA, decursinol, nodakenin and nodakentin to treat cervical cancer. They reported that decursin and decursinol successfully inhibited cervical cancer cells’ growth via induction of tumor necrosis factor-related apoptosis-induced ligand (TRAIL). In this process, several anti-apoptosis proteins including Bclx, Bcl2, clapsin, survivine and clusterin were downregulated, while simultaneously; pro-apoptotic proteins like caspase-3, DR-4 and DR-5 TRAIL receptors were upregulated. Experimental analysis of HeLa cells therefore elucidated that decursin and decursinol are potentially active inhibitors of cervical cancer. However, there were no negative effects on normal cells.

### 6.3. AGNACCs’ Action Mechanism in Prostate Cancer

Another study [6] reported that primary prostate cancer cells’ (RC-58T/h/SA#4) proliferation was successfully inhibited by AGN’s active compound decursin in a dose-dependent manner. The authors proposed that proliferation of RC-58T/h/SA#4 cells was inhibited through apoptosis by mitochondrial caspase-dependent and -independent pathways. They showed that decursin induced an extrinsic apoptosis process, activated caspase-8 and caused cleavage of the Bid protein, which is involved in regulating the apoptosis process. Thus, pro- and anti-apoptotic proteins were imbalanced, leading to upregulation of Bax proteins and downregulation of anti-apoptotic proteins. The variable proportion of Bax and Bcl2 proteins caused release of cytochrome c that further interacted with caspase-9 and ATP-dependent proteolysis factor [APF] and continued with intrinsic apoptosis. The intrinsic apoptosis ultimately led to Poly [ADP-ribose] polymerase [PARP) cleavage. Additionally, decursin was found to be induced via a caspase-independent pathway, and furthermore, induced Apoptosis inducing factor [AIF] and nuclease G-protein expression was studied by Western blot. The upregulated proteins translocated into the nucleus, causing chromatin condensation and large-scale DNA fragmentation. Their studies have shown evidence that decursin could induce caspase-dependent and -independent pathways to potentially inhibit growth of prostate cancer cells.

### 6.4. AGNACCs’ Action Mechanism in Melanoma

**Figure 4:**
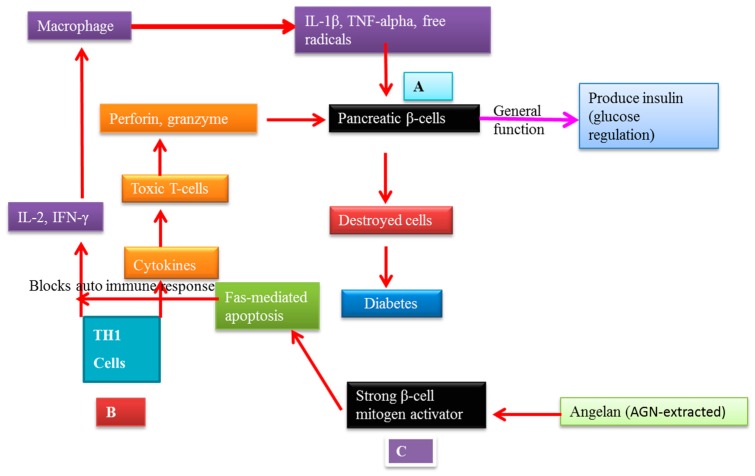
Angelan’s molecular action mechanism against autoimmune diabetes. (**A**) Pancreatic β-cells’ general function is glucose regulation in blood by producing insulin; (**B**) Autoimmune systems destroy the pancreatic cells, losing insulin-producing ability, ultimately leading to diabetes; (**C**) Angelan is a strong mitogen activator that can block the autoimmune response by Fas-mediated apoptosis.

Kim et al. elucidated the molecular action mechanism of decursin in B16F10 melanoma cells. In-vitro and in-vivo experiments were performed using B16F10 cells as well as in the mouse as a model system. Calcium-dependent phospholipid-binding proteins (annexins) were stained to detect the morphological and molecular changes in the apoptosis process. The results showed shrunken cells, fragmented DNA and cytoplasm condensation in melanoma cancer cells, which affirmed the apoptosis process. Both the extrinsic and intrinsic apoptosis pathways pass through caspase-3, and their Western blot analysis also confirmed caspase-3 activation in a time- and dose-dependent manner. An in-vivo experiment was carried out with implanted melanoma cancer cells into C57BL6 mice. The AGN extraction (10 mg/kg) was injected intraperitoneally for 2–3 weeks on every alternate day after tumor implantation. Further tumor growth was checked for three weeks. The tumor weight significantly decreased as compared to the control. The results elucidated that decursin was successful in inhibiting the proliferation of melanoma cancer cells without any negative effect on normal cells [50]. Another study also revealed that one of the AGNACCs’ pectic polysaccharides, angelan, was successful in inhibiting melanoma by boosting the immune system with strong mitogen activity. Through the induction of NF-κB/Rel, an increase in expression levels of iNOS, IL-1β and tumor necrosis factor α was observed, and this resulted in prolonged lifespan of melanoma-implanted mice [51]. The mechanism, depicted in Figure 4, is similar to autoimmune diabetes inhibition.

### 6.5. AGNACCs’ Action Mechanism in Bladder and Colon Cancer

Decursin’s role in the cancer cell cycle and signaling pathways was elucidated by Kim et al. [52] by using bladder cancer 235J cells and colon cancer HCT116 cells. The MAP kinase family proteins are the crucial players in cell-cycle regulation; they include extracellular signal-related kinase (ERK), C-Jun N-terminal Kinase(C-JNK) and p38. The study clearly explained that decursin was activated by extracellular signal-related kinase (ERK kinase), which was induced at G1 cell-cycle arrest via p21WAF1 in cancer cells. To prove the role of ERK kinase in cell proliferation inhibition, pharmacological inhibitor PD98059 treatment was given prior to decursin administration, resulting in reduced expression of p21WAF1. Thus, it was clearly evident that ERK played a crucial role in cell proliferation inhibition. They showed that dose-dependent decursin induced apoptosis in cancer cells with decreased cell viability, and increased G1-phase cell numbers. Cell-cycle regulatory factors like cyclin D1, E, CDk2 and CDk4 were examined with decursin (50–100 μM). The results showed reduced protein expression levels in a dose-dependent manner. The study also elucidated the mechanism of apoptosis events, which were induced by decursin treatment, including cytochrome c release, caspase-3 activation, Bax protein upregulation and Bcl2 protein downregulation in both cells. The molecular action mechanism of decursin in cell-cycle regulation was thus explored. 

#### Lung Cancer and Sarcoma

In-vivo experiments were conducted to screen pyranocoumarin compounds’ efficacy with the Lewis lung cancer mouse model. 4 mg/kg of decursin was injected intraperitoneally for 21 days. The results showed that the tumor growth and microvessel density were significantly reduced in the decursin-treated group as compared to the control group. They explained that a possible reason for angiogenesis suppression was that the extrinsic apoptosis might be involved in this process through suppression of p-REK, p-JNK and phospho-VEGFR [53].

In-vivo experiments with ICR mice were conducted for sarcoma-180 tumor-suppression analysis with D and DA in 50 and 100 mg/kg doses for nine days of consecutive intraperitoneal injection. The results clearly showed that the allo-grafted mice lifespan was extended with D and DA treatment as compared to control mice. The median lifespans of the 50 and 100 mg/kg DA-treated groups were 32.3 and 34.2 days, respectively, and the D-treated group lifespans were 29 and 39 days with the respective treatment regimes; the control was 22.4 days [54].

### 6.6. AGNACCs’ Action Mechanism in Sexual Hormone-Dependent Cancers

Prostate cancer is mainly caused by androgen, whose receptors are the major targets for prostate cancer prevention [55,56]. Although hormone-ablation therapy is efficient for androgen receptor AR-dependent prostate cancer prevention, the AR-independent pathway also causes prostate cancer [57]. Hence, there is a need to focus on new agents that are responsible for inhibition of AR-dependent and -independent progression of prostate cancer. 

Guo et al. found natural inhibitor activities from AGNACCs for AR-dependent and -independent prostate cancer prevention in LNCaP cells. Their experimental results showed that decursin was an effective AR inhibitor and also inhibited nuclear AR translocation, prostate-specific antigen (PSA) suppression, and decreased AR protein quantity. The experiment also proved that the natural D and DA compounds were potentially more effective than synthetic androgen inhibitor bicalutamide, due to the latter’s discriminate, agonistic activity in the absence of androgen. They showed mechanistically the structural relationships and action mechanism of D, DA and decursinol. Their results clearly explained that the side chain of the pyranocoumarin ring is crucial for apoptosis, and its anti-AR effect reduced AR content via 26S proteasomal degradation. Reactive oxygen species (ROS) induction and PSA inhibition was observed in a dose-dependent manner. Decursin, especially, was more effective than decursinol in suppression of dihydrotestosterone’s (DHT’s) adhesion to the androgen receptor. Decursinol exhibited a biphasic nature; it was suppressed by PSA at lower concentrations, whereas at higher concentrations, PSA mRNA expression was upregulated through stimulation of its upstream region. The elevated concentration did not affect the prostate cancer cells (DU145 and PC-3 cells) that were lacking androgen receptors [14].

The same group simultaneously screened decursin and decursinol angelate’s efficacy on human breast cancer cells, mediated by estrogen-dependent and -independent cells MCF-7 and MDA MB-231, respectively. They showed similar results wherein they found that the pyranocoumarin side chain is crucial for cellular apoptosis, which means that D and DA were more effective than decursinol. More than 20 µM D and DA concentrations were effective in suppression of the breast cancer cell (MCF-7 cell) growth through estrogen receptor-α suppression, whereas estrogen receptor-β suppression inhibited MDA MB-231 growth. Here also, they showed that D and DA were more effective than the breast cancer chemopreventive drug tamoxifen, because they were not agonistic in the absence of estrogen. They concluded that D and DA were effectively suppressing cell growth in breast cancer cells by arresting the G0–1 phase and inducing the apoptosis process [1]. Another study [58] showed that the increased cytosolic β-catenin was degraded due to the effect decursin inhibition of AR-independent prostate cancer.

Two decades ago, Ahn et al. [59] investigated the effect of AGNEtOH against erythroleukemia K562 cells and reported that the anticancer property was associated with protein kinase activation [60]. Further investigation by the same group revealed that D and DA were cytotoxic and responsible for protein kinase C PKC activation [60,61]. Another study elucidated the major differences in the mechanisms of action between D and phorbol 12, 13 dibutyrate (PDBu), two PKC activators, in human erythroleukemia cells (K562) [62]. They noticed quite opposite results with D and PDBu in bleb formation and megakaryocytic differentiation. PDBu was quickly promoted the bleb formation as well as tumor growth, whereas decursin negatively regulated PDBu’s effect [62]. Another group conducted experiments with myeloleukemia (U937) and erythroleukemia cells (K562) and unraveled the involvement of two PKC activators and ROS (reactive oxygen species) in negative cell modulation of D and DA [63]. They revealed that the 12 derivatives of decursin activated PKC in the presence of phosphatidylserine (PTS), whereas decursinol activated PKC even in the absence of PTS. Both D and DA showed cytotoxic effects on K562, U937 and TUR cells. Additionally, the authors showed that the structure of decursin was vital for anticancer effects and that the side chain was crucial for PKC activation and negative modulation of the leukemia cells [63].

Another study also supported the hypothesis that decursin may act as a negative modulator in myeloid leukemia KBM-5 cells through the induction of the apoptosis process via downregulation of cyclooxygenase-2 and survivine [64]. Kim et al. [65] explained that decursin was effective in suppressing several multiple-myeloma cell lines (U266, MM.1s and ARH77) by suppression of JAK2/STAT3, cyclin D1 and bcl2.

Another study reported that combined treatment with AGN-derived decursin and doxorubicin was effective in enhancing the apoptotic pathway via targeting of rapamycin mTOR and sTATE3 signaling pathways in multiple-myeloma cells (U266, RPMI8226 and MM.1s cells). They suggested that a combined effect of decursin and doxorubicin was more efficient than treating with either compound alone. Furthermore, treatment enhanced the activation of caspase-9 cleavage of PARP, reduced mitochondrial membrane potential and suppressed phosphorylation of JAK2 and STAT3 [66].

Recently, Shehazad et al. [67] reported that DA was an effective inhibitor of prostaglandin E2 (PGE_2_)-induced survival of the human leukemia HL-60 cell line via the regulation of the EP2 receptor and NFκB pathway. They showed previously that PGE_2_ can enhance HL60 cell survival by protecting them from the induction of apoptosis through oxidative stress. Furthermore, their study revealed that DA can effectively suppress the PGE_2_ effect, and thus the PGE_2_ effect was nullified, which blocked activation of PGE_2_-induced COX-2 and EP2 Ras/Raf/Erk pathways. Their results conclusively elucidated that DA was effective against the PGE_2_-induced anti-apoptotic process by the regulation of the EP2 receptor and NfkB pathway [67].

## 7. Other Biological Activities 

### 7.1. AGNACCs’ Molecular Mechanism in Neuroprotective Effects

Acetylcholine is a very crucial organic chemical for neurotransmission in the brain. It is also known as cholinergic in medical terminology. Cholinergic neurons are important for memory and their damage leads to memory impairment or senile dementia [68]. Several attempts were made for regaining cholinergic activity by an acetylcholine precursor, acetylcholinesterase suppression or cholinergic agonists; however, the best method found was cholinesterase inhibition [69]. The Korean medicinal herb *A. gigas* contains coumarin compounds that constitute effective anticholinesterase activity [70]. Methanol-extracted AGN decursin was a major compound that ameliorated scopolamine-induced memory impairment [71]. Although decursinol showed a great effect on scopolamine-induced amnesia, the majority of the experiments were conducted with decursin. Kim et al. [66] described that nodakenin, a derivative of *A. gigas*, also showed effects similar to that of decursin. Neuron damage from glutamate-induced neuron injury was successfully minimized by decursinol and decursin pretreatment in rat cortical cells [72]. The authors explained that the glutamate-induced intracellular Ca^2+^ influx was significantly reduced by decursin and decursinol glutathione activity.

Senile dementia, also known as Alzheimer’s disease (AD), is predominantly affecting millions of the people on the globe [73]. Senile plaques are extracellular depositions of β (Aβ)-amyloid in the brain, and they directly induce neural apoptosis, ultimately leading to Alzheimer’s disease (AD). Aβ1-42-induced memory impairment was effectively protected by long-term administration of AGN’s ethanolic extraction of decursinol [74]. Another study [75] showed that the AGN standard ethanolic extract, INM-176, was successful in mitigating lipopolysaccharide-induced cognitive dysfunction. Furthermore, their investigation elucidated INM-176’s effect on scopolamine or Ab1-42 protein-induced memory impairment [64]. The study explained the increased astrocytes density in the hippocampus post-Aβ1-42 administration in rats in contrast to inhibition of Aβ1-42-induced inflammation treated with INM-176. They presumed that INM176 may ameliorate the cognitive dysfunction through cholinergic neurotransmission modulation [75]. All of the above studies well-explained the protective effects against neurodiseases, but none of the studies elucidated the mode of action or mechanism. Another group of researchers showed that AGN–decursin was effective in protecting neural cells (PC12 cells) against Aβ-induced oxidative damage through NrF2 transcription factor-mediated heat-shock protein 32 activation [2]. Furthermore, the same group extended the research on PC12 cell lines to elucidate the possible mechanism of Aβ-induced memory impairment. Deposition of Aβ in the cell causes excessive production of ROS which can lead to mitochondrial dysfunction. Mitochondrial therapy is considered as an effective method for treating AD [76]. Mitochondrial therapy might play a crucial role in controlling AD, which was shown in this study. Mitochondria apoptosis pathways are regulated by anti-Bcl-2 and pro-Bax proteins. Elevation of Aβ deposition results in Bax protein upregulation and Bcl-2 protein downregulation, resulting in ROS overproduction, which further leads to mitochondria dysfunction. Pretreatment of PC12 cells with decursin was effectively downregulated pro-apoptosis Bax proteins. These results elucidated an AD control mechanism, that is, via mitochondrial therapy by AGN-derived decursin pretreatment [77]. AGN flavonoid extractions, especially the hexane fraction, promoted the growth and extension of pheochromocytoma (PC12) cells. In addition, they reported that PC12 cell apoptosis was greatly inhibited by suppressing DNA damage, particularly in the presence of major flavonoids quercetin, myricetin and catechin [78]. 

### 7.2. AGNACCs’ Action Mechanism in Autoimmune Diabetes

By using pectic polysaccharide angelan extracted from AGN, Kim et al. [10] showed an inhibitory effect on autoimmune diabetes by suppressing autoimmunity in non-obese diabetic (NOD) mice. Angelan was administered intraperitoneally with 30 mg/kg on every alternate day from eight to 24 weeks of mouse age. None of the treated mice were affected by diabetes, whereas eight out of 10 control mice exhibited diabetes. Late-stage (15–10 weeks) treatment with angelan delayed diabetes progression, which indicated that even after insulitis initiation, angelan was effective. They screened angelan antidiabetic activity regarding regulation of cell autoreactivity. Angelan-treated NOD spleen cells were transfused into six-week-old NOD. *Scid* cells. All the mice became diabetic within seven weeks after implantation, whereas diabetes was delayed in angelan-treated spleen cells until nine weeks. They explained that the emerged diabetes in NOD mice caused by angelan autoreactivity destroyed β-cells. Angelan (30 µg/kg) did not protect β-pancreatic cells when they used a streptozotocin chemical-based destruction. Even though the results did not clearly explain the molecular mechanism, their transfusion experiment analysis suggested that angelan could regulate autoreactive immune cells, indicating that the cells did not encounter autoantigens of β-cells. The proposed molecular mechanism of decursin is depicted in Figure 4.

### 7.3. AGNACCs’ Anti-Inflammatory Activity

Inflammation is a useful host response to the pathogen’s harmful stimuli that helps in protection from diseases [79]. The defense mechanism is activated through the host immune response by NADPHoxide-induced nitric oxide synthase (iNOS) and other cytokines. NADPH-oxide and iNOS produce O^2^^−^ and nitric oxide in macrophages and the cytosol, respectively [80,81]. NO is a crucial messenger in the defense response against a wide range of pathogens [82]. Production of elevated levels of NO is cytotoxic to the cell itself, and thus, tight regulation of NO is essential [83]. The complications raised through inflammation may lead to severe diseases that are associated even with cancer. NO production regulation is controlled by heme oxygenases (HOs) that can catalyze the deterioration of heme. Heme oxygenase is a vital enzyme for maintaining the cellular homeostasis to combat ROS [84,85]. It has been already documented that HO-1 was induced by NO production, and it showed anti-inflammatory activity by inhibiting NO production [86,87].

**Figure 5:**
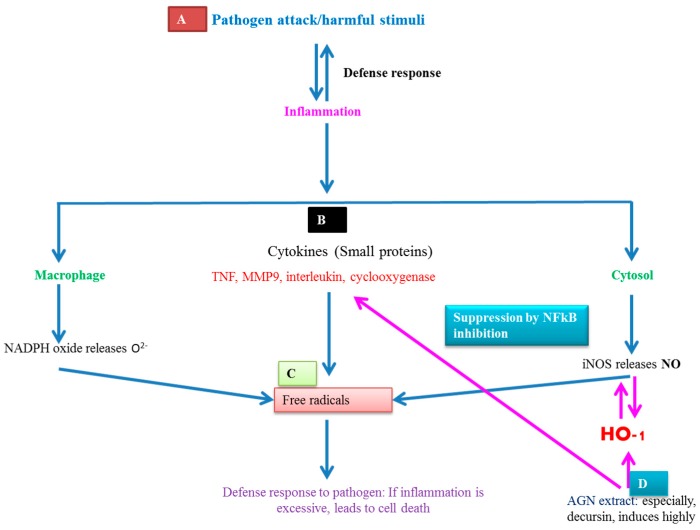
Mechanism of action of the major active pyranocoumarin, decursin, in the process of anti-inflammation. (**A**) Pathogens’ harmful stimuli induce host immune system and host beneficial responses, inflammation process activated; (**B**) Cytokines like small proteins, generates free radicals which leads to oxidative damage can prevented by AGNACCs; (**C**) Due to the inflammation, NADPH-oxide releases O^2−^ in macrophages and in the cytosol, nitric oxide released from iNOS (induced nitric oxide synthase). Overproduction of the inflammatory messengers results in pathogen death as well as cell death; (**D**) Deursin induces HO-1 (heme oxygenase) that could regulate nitric oxide, thus the inflammation process will be controlled. Decursin also inhibits NF-κB (nuclear factor kappa B) that ultimately regulates inflammation through cytokine regulation.

Nuclear factor κB (NF-κB) is a key activator of proinflammatory cytokines such as matrix metallopeptidase-9 (MMP-9) and iNOS. Thus MMP-9 and iNOS produce NO, which may lead to inflammatory complications. Kim et al. [35] showed that decursin derived from AGN leads to inhibition of the transcriptional activation of these genes, which indicates an anti-inflammatory role of decursin. In another study, ethanol-extracted coumarins effectively inhibited iNOS and other cytokines such as interleuken-1β and COX-2 that are responsible for inflammatory activities [82]. Cho et al. [12] showed that the AGN extract containing D, DA and nodakenin was effective in suppressing nitric oxide overproduction. Hence, the authors indicated an anti-inflammatory role of AGN extract through the induction of HO-1. They showed that even in the absence of CoPP (an HO-1 activator), HO-1 was greatly expressed when treated with coumarins derived from AGN. The molecular action mechanism of AGNCCSs is shown in Figure 5.

## 8. Conclusions

*Angelica gigas* Nakai is naturally found in Korea and has a higher concentration of D and DA as compared to other Chinse and Japanese dang-gui. Cancer has no specific medicine except for some therapies like chemo or immune therapy, which carry severe side effects. Thus, researchers are focusing on new phytochemical-mediated remedies. Numerous attempts were made to decipher the therapeutic roles of D and DA in cancer, and their other biological activities. AGNACCs D and DA have therapeutic potential against cancer, and possess other biological activities, through the induction of programmed cell death. Although a few reviews were published on D and DA, elucidating their role in cancer and their other biological activities, none of the publications elucidated their molecular action mechanism. Here, we have explained the biosynthesis of AGNACCs, their effective extraction and formulation methods, and their molecular action mechanism in cancer and in other biological activities. The in-vitro and in-vivo experimental studies showed positive results without any adverse effects; however, most of the experimental trials were done with rodents, and very few trials were done with humans. Thus, further trials are needed to identify the therapeutic roles of AGNACCs in other diseases.
